# Research hotspots and emerging trends of macrophages in peripheral nerve injury: a bibliometric and visualization analysis

**DOI:** 10.3389/fncel.2026.1893849

**Published:** 2026-09-17

**Authors:** Guohua Jiang, Renkun Huang, Kewen Wang, Yuchang Gui, Yun Tang, Guiyang Jiang, Yijun Liu, Jianwen Xu

**Affiliations:** 1 Department of Rehabilitation Medicine, The First Affiliated Hospital of Guangxi Medical University, Nanning, Guangxi, China; 2 Department of Foot and Ankle Surgery, Center for Orthopedic Surgery, The Third Affiliated Hospital of Southern Medical University, Guangzhou, China

**Keywords:** bibliometric analysis, immunomodulation, macrophage, nerve regeneration, peripheral nerve injury

## Abstract

**Objective:**

Peripheral nerve injury (PNI) remains a significant clinical challenge due to its poor neurological recovery. Macrophages serve as key regulators in pathological degeneration, inflammatory resolution, and regenerative repair after PNI. This study aims to explore the global status, research hotspots, and developmental trends of macrophage-related studies in PNI through bibliometric analysis. It further aimed to identify emerging research frontiers and existing knowledge gaps, thereby providing insights into optimized PNI repair strategies.

**Methods:**

Literature related to PNI and macrophages published from January 1, 2006, to March 31, 2026, was retrieved from the Web of Science Core Collection and Scopus databases. After literature screening, deduplication, and data standardization, VOSviewer, CiteSpace, and Bibliometrix were used to perform bibliometric analysis and visualization of countries, institutions, authors, journals, keywords, and citations.

**Results:**

A total of 694 publications were included. The annual publication output showed a continuous upward trend, with a marked increase after 2017 and a peak of 76 publications in 2025. A total of 50 countries contributed to this field. China had the highest number of publications, whereas the United States had the highest total number of citations. Nantong University was the most productive institution, and Kiguchi, Norikazu and Kishioka, Shiroh were the most productive authors. *Journal of Neuroinflammation* was the leading journal in this field. Macrophage polarization, Wallerian degeneration, neuropathic pain, macrophage–Schwann cell interactions, immunomodulation, and inflammatory microenvironment remodeling were the long-standing core hotspots in this field. In recent years, subcellular injury and neural excitability mechanisms, including apoptosis and efferocytosis, mitochondrial dysfunction, and ion channels, have received increasing attention. Tissue engineering and biotherapeutic strategies, such as nerve conduits, mesenchymal stem cells, exosomes, and drug delivery, have become emerging research directions. Moreover, the application of transcriptomics and single-cell RNA sequencing has further advanced the in-depth analysis of macrophage heterogeneity and molecular regulatory mechanisms.

**Conclusion:**

This study revealed the hotspots and trends of macrophage research in PNI over the past two decades. Research foci have expanded from basic pathological mechanisms to neuroimmune regulation and regenerative strategies. Combined omics and bioinformatics will help elucidate macrophage regulatory networks and advance precise interventions for PNI.

## 1 Introduction

Peripheral nerve injury (PNI) is a common type of neurological injury in clinical practice. It is often caused by trauma, compression, traction, or iatrogenic injury, and frequently results in sensory impairment, motor dysfunction, and neuropathic pain, thereby severely reducing patients' quality of life and increasing the medical burden (Bage and Power, 2021; Randall et al., 2026). Although the peripheral nervous system has a greater regenerative capacity than the central nervous system, functional recovery after severe injury remains unsatisfactory. This is particularly evident in cases of long-distance nerve defects, proximal nerve injuries, and delayed repair, in which slow axonal regeneration, denervation atrophy of target organs, and scar formation often compromise the final repair outcome (Li D. D. et al., 2022). Therefore, elucidating the key mechanisms that limit nerve regeneration after PNI and identifying effective therapeutic targets have become important research directions in the field of peripheral nerve repair.

The pathological repair process after PNI involves multiple events, including Wallerian degeneration, inflammatory responses, myelin debris clearance, Schwann cell activation, axonal regeneration, and remyelination. Among these processes, the immune microenvironment plays a crucial regulatory role in injury progression and regenerative repair (Bosch-Queralt et al., 2023). As one of the most important immune cell populations after injury, macrophages not only participate in the clearance of myelin and axonal debris, but also regulate the local microenvironment by secreting inflammatory factors, chemokines, and growth factors, thereby influencing Wallerian degeneration, axonal regeneration, and functional recovery (Li Y. et al., 2022; Xia et al., 2025). In recent years, increasing attention has been paid to the mechanisms and intervention strategies related to macrophages in PNI. However, these studies are distributed across multiple disciplines, including neuroscience, immunology, pain medicine, and regenerative medicine, and the research topics remain relatively scattered. A systematic overview of the overall research status, knowledge structure, and hotspot evolution in this field is still lacking.

Bibliometrics is an interdisciplinary research method based on statistics and documentation. It evaluates publication trends, country and institutional collaborations, author distribution, journal sources, keywords, and citation relationships. Through these analyses, bibliometric approaches can reveal the knowledge base, research hotspots, and frontier evolution of a specific field (Donthu et al., 2021). Compared with traditional narrative reviews, bibliometric analysis provides a more systematic representation of the developmental trajectory of a research field, especially in areas characterized by rapidly increasing publications and complex interdisciplinary topics. Although previous reviews have summarized the role of macrophages in peripheral nerve injury repair (Liu et al., 2019), a systematic bibliometric analysis focusing specifically on macrophage-related research in PNI is still lacking, making it difficult to comprehensively understand the research landscape and emerging directions from a macro-level perspective. Therefore, based on the Web of Science Core Collection and Scopus databases, this study used bibliometric and visualization tools, including VOSviewer, CiteSpace, and Bibliometrix, to systematically analyze macrophage-related publications in PNI from January 2006 to March 2026. This study aimed to clarify the global research status, hotspot themes, and evolutionary trends in this field, thereby providing a reference for future mechanistic investigations and the development of therapeutic strategies for peripheral nerve repair.

## 2 Materials and methods

### 2.1 Data sources and inclusion/exclusion criteria

This study strictly followed the PRISMA 2020 guidelines for literature screening and data extraction. To ensure the authority, comprehensiveness, and retrieval stability of the literature data, two major international academic databases, Web of Science Core Collection (WoSCC) and Scopus, were selected as data sources. These databases provide complementary literature coverage and standardized bibliographic and citation metadata that can be reliably exported and analyzed using commonly used bibliometric software. WoSCC provides highly curated citation indexing, whereas Scopus offers broader journal coverage. Therefore, combining these two databases can improve literature coverage while maintaining the consistency of bibliographic data required for citation analysis, network construction, and knowledge mapping.

The literature search was completed at the same time to avoid potential bias caused by real-time database updates. The search period was set from January 1, 2006, to March 31, 2026. The inclusion criteria were as follows: (1) studies directly related to peripheral nerve injury and macrophages; (2) publication types limited to original articles and reviews; and (3) publications with complete bibliographic information and accessible full texts. Publications were restricted to English because the bibliometric workflow relied on standardized titles, abstracts, author keywords, and indexed keywords, and the use of a single language reduced heterogeneity introduced by multilingual terminology during data cleaning and keyword normalization. The exclusion criteria were as follows: (1) non-English publications; (2) conference abstracts, conference papers, case reports, letters, retracted articles, book chapters, and pre-prints; and (3) publications whose content was unrelated to the research topic.

### 2.2 Search strategy and screening process

The search strategy used in the Web of Science database was as follows: TS=(“peripheral nerve injury” OR “peripheral nerve lesion” OR “peripheral nerve trauma” OR “PNI”) AND TS=(“macrophage” OR “macrophagocyte” OR “macrophagus” OR “megalophage”) AND DT=(Article OR Review) AND LA=(English). Publication years: January 1, 2006–March 31, 2026.

The search strategy used in the Scopus database was as follows: TITLE-ABS-KEY(“peripheral nerve injury” OR “peripheral nerve lesion” OR “peripheral nerve trauma” OR “PNI”) AND TITLE-ABS-KEY(“macrophage” OR “macrophagocyte” OR “macrophagus” OR “megalophage”) AND DOCTYPE(ar OR re) AND LANGUAGE(English) AND (PUBYEAR > 2005 AND PUBYEAR < 2027). The final search was conducted on March 31, 2026, and only publications available up to this date were included.

First, two independent researchers, Guohua Jiang and Renkun Huang, screened the titles and abstracts of the retrieved records and excluded publications that were clearly unrelated to the topic or did not meet the publication type criteria. Subsequently, the full texts of the initially retained publications were reviewed, and eligibility was determined strictly according to the pre-defined inclusion and exclusion criteria. Inter-reviewer agreement was assessed on the basis of the independent screening decisions before adjudication using Cohen's kappa coefficient. The Cohen's κ value was 0.85, indicating almost perfect agreement between the two reviewers. Any disagreement between the two researchers was resolved by a third senior researcher, Kewen Wang, who conducted an additional review and made the final decision. The complete literature screening process is shown in Figure 1.

**Figure 1:**
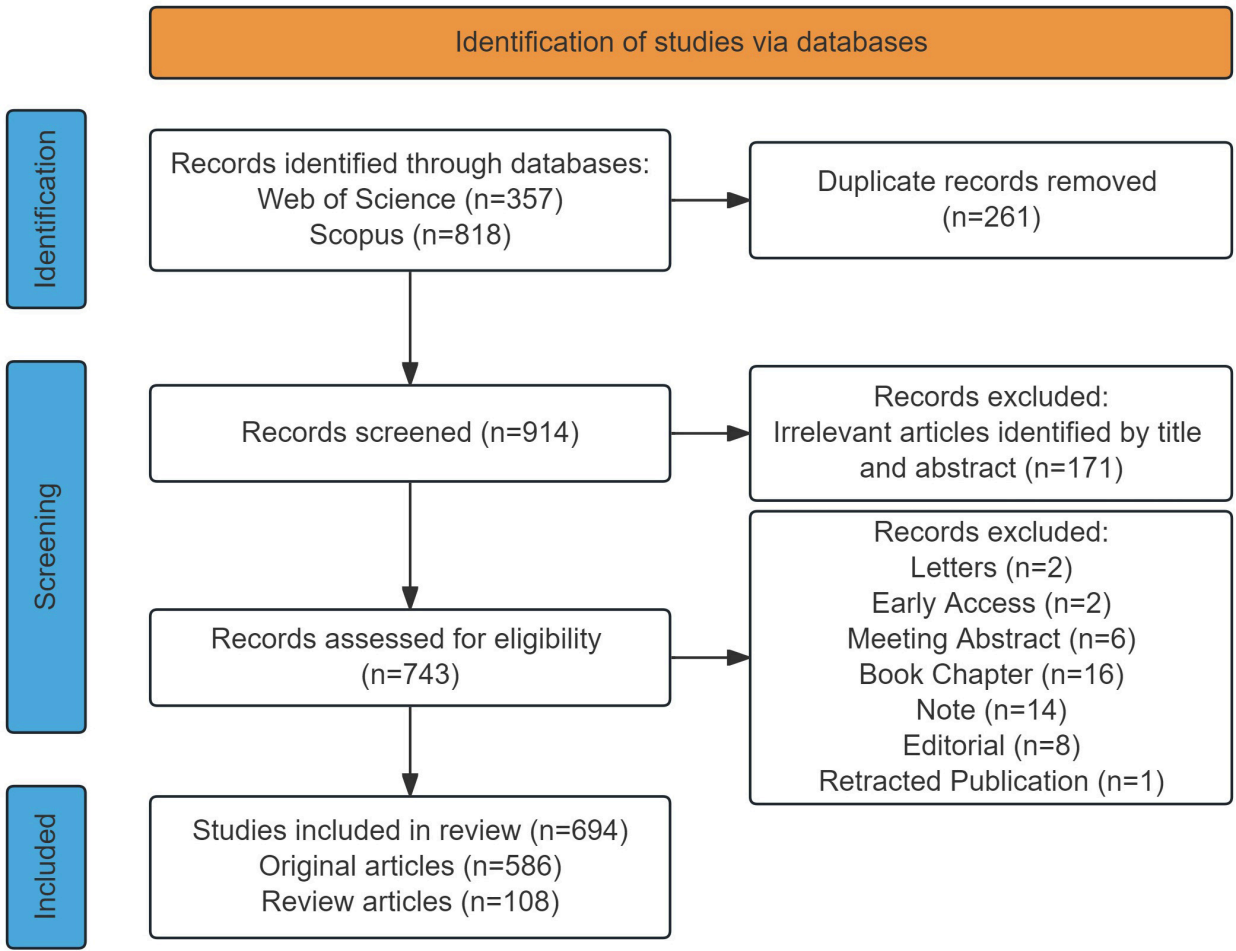
Flowchart of the literature screening process.

### 2.3 Data standardization

VOSviewer was used to identify country/region names and keywords in the included publications. Data cleaning and standardization were performed using the merge and replacement functions of the thesaurus file, as follows:

(1) Standardization of country/region names: first, internationally accepted names were used to standardize country names. For example, “Peoples R China” was standardized as “China,” and “United States” was simplified as “USA.” Second, regional names were assigned to their corresponding countries. For example, “Scotland” and “England” were unified under “United Kingdom” to ensure consistency and accuracy of geographic information.
(2) Standardization of keywords: first, synonymous keywords were merged according to commonly accepted disciplinary terminology. For example, “immunoregulation” and “immunomodulation” were merged as “immunomodulation,” and “sciatic nerve crush” and “sciatic nerve injury” were unified as “sciatic nerve injury.” Second, different expressions with the same core meaning were standardized. For example, “sprague dawley rat” was standardized as “rat,” and “spinal-cord” was unified as “spinal cord.” Third, singular and plural forms were standardized. For example, “macrophages,” “hydrogels,” and “mice” were standardized as “macrophage,” “hydrogel,” and “mouse,” respectively. All merging and modification records for country/region names and keywords are provided in Supplementary Table S1.

### 2.4 Visualization tools and analysis

To systematically and comprehensively perform bibliometric analysis and knowledge mapping, multiple widely used bibliometric tools were integrated for multidimensional data analysis. VOSviewer (version 1.6.20) was used to construct collaboration network maps of countries/regions, institutions, authors, and journals, as well as keyword co-occurrence network maps. The minimum number of documents was set according to the specific analysis objects. Specifically, the thresholds were set as follows: countries (minimum number of documents = 1), institutions (minimum number of documents = 5), authors (minimum number of documents = 5), journals (minimum number of documents = 4), and keywords (minimum number of occurrences = 21). CiteSpace (version 6.4.R1) was used to generate dual-map overlays of journals, analyze co-cited references, and identify burst keywords and highly cited references with strong citation bursts through burst detection, thereby capturing the evolution of research hotspots and frontier trends. The parameters were set using a standard time-slicing mode of 1 year per slice, with the g-index (*k* = 25) as the node selection criterion, and the Pathfinder pruning algorithm was applied to optimize the network structure. For citation burst detection, the minimum duration was set to 2 years, and the burst detection model parameters were configured according to the default settings of CiteSpace. Scimago Graphica (version 1.0.53) was used to generate global geographic distribution maps and country collaboration maps. R software with the Bibliometrix package (version 5.0) was used for systematic statistical and quantitative analyses of institutional contributions, thematic evolution, and other bibliometric indicators, thereby assisting in identifying the overall developmental characteristics of the field. Microsoft Office Excel 2019 was used to analyze annual publication trends, summarize raw data, and construct statistical tables. The complete parameter settings for VOSviewer and CiteSpace analyses are provided in the Supplementary material “Software_Analysis_Parameters.”

## 3 Results

### 3.1 Analysis of publication trends

A total of 694 publications were ultimately included in this study, comprising 586 original research articles and 108 reviews. The annual and cumulative publication trends from January 2006 to March 2026 are shown in Figure 2. Overall, the annual number of publications related to macrophages in peripheral nerve injury showed a steady upward trend, with only minor year-to-year fluctuations. The number of publications started at 16 in 2006. Although transient decreases were observed in 2009 and 2016, the field entered a phase of rapid growth after 2017, increasing to 54 publications in 2020 and reaching an annual peak of 76 publications in 2025. In the first quarter of 2026, 24 publications were identified, suggesting that the total annual output may exceed 90 publications. Exponential fitting of the cumulative publication volume yielded the equation *y* = 0.9831e^0.6786x^, with a coefficient of determination of *R*^2^ = 0.9955, indicating an excellent fit and confirming a stable exponential growth trend in the total number of publications in this field. This growth trajectory indicates that research on the pathological and reparative roles of macrophages in peripheral nerve injury has attracted increasing global academic attention and still has considerable potential for future development.

**Figure 2:**
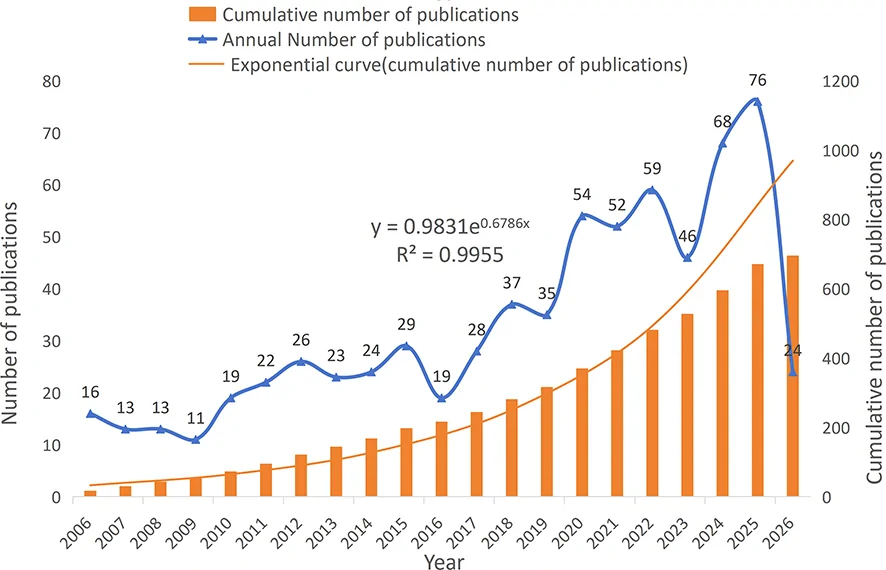
Annual and cumulative publication trends in research on macrophages in peripheral nerve injury from January 2006 to March 2026.

### 3.2 Analysis of countries and institutions

From January 2006 to March 2026, researchers from 50 countries conducted studies on the role of macrophages in peripheral nerve injury. Scientific output in this field was mainly concentrated in Asia, North America, and Europe, and the global geographic distribution is shown in Figure 3A. Among the top 10 most productive countries (Table 1), China ranked first with 263 publications, followed by the United States with 202 publications and Japan with 62 publications. These countries constituted the core contributors to this field. In terms of citation indicators, the United States had the highest total number of citations (13,661), followed by China (7,679). The average citation analysis showed that Canada, the United Kingdom, and the United States had higher citation impact per publication, reflecting the high quality and broad academic recognition of research from these countries. The country collaboration network maps (Figures 3B, C) showed that an international collaboration pattern centered on China and the United States has formed in this field, with the strongest collaborative link observed between these two countries. Extensive academic cooperation was also observed among developed countries in Europe and North America. Although some countries, such as Poland, Austria, and Romania, had a certain level of research output, they had fewer international links and lower total link strength, suggesting that cross-regional academic exchange needs to be further strengthened.

**Figure 3:**
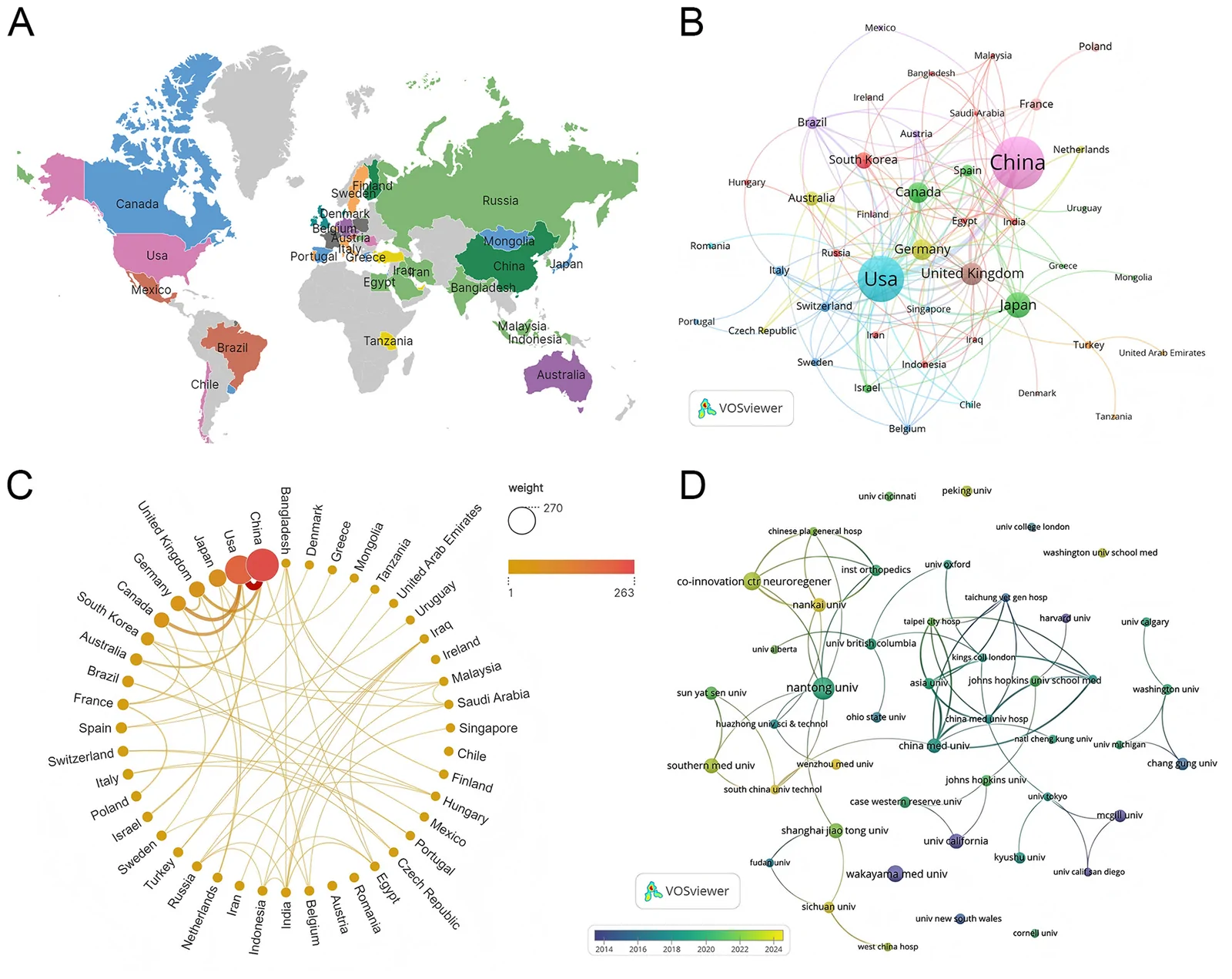
Visualized analysis of countries and institutions in research on macrophages in peripheral nerve injury. **(A)** Global geographic distribution map of countries. **(B)** Country collaboration network generated using VOSviewer. **(C)** Circular diagram of country collaboration. **(D)** Institutional collaboration network generated using VOSviewer.

**Table 1:** Top 10 most productive countries in research on macrophages in peripheral nerve injury.

Rank	Country	Publications	Citations	Average citations	Total link strength
1	China	263	7,679	29.2	57
2	USA	202	13,661	67.6	100
3	Japan	62	2,512	40.5	21
4	United Kingdom	47	3,912	83.2	46
5	Canada	42	3,592	85.5	31
6	Germany	42	2,747	65.4	39
7	South Korea	26	1,114	42.8	10
8	Australia	20	1,108	55.4	17
9	Brazil	16	722	45.1	16
10	France	14	612	43.7	11

The top 10 most productive institutions are listed in Table 2. Nantong University ranked first globally with 24 publications, followed by the Co-Innovation Center of Neuroregeneration with 17 publications and Wakayama Medical University with 15 publications. Among the top 10 institutions, eight were Chinese research institutions, highlighting China's strong research foundation and productivity in this field. The institutional collaboration network (Figure 3D) showed a clear pattern of regional clustering. Collaborations among core Chinese institutions were close and densely connected, particularly within the internal collaboration network centered on Nantong University, the Co-Innovation Center of Neuroregeneration, Southern Medical University, and Shanghai Jiao Tong University. In contrast, cross-national institutional collaborations were relatively sparse. China Medical University established only a limited number of cross-national or cross-regional collaborative links with Asia University, Johns Hopkins University School of Medicine, and King's College London, while other highly productive Chinese institutions had relatively few international collaborations. These findings suggest that deeper cross-national institutional collaboration should be further strengthened in the future.

**Table 2:** Top 10 most productive institutions in research on macrophages in peripheral nerve injury.

Rank	Institution	Publications	Citations	Average Citations	Country
1	Nantong University	24	576	24	China
2	Co-Innovation Center of Neuroregeneration	17	323	19	China
3	Wakayama Medical University	15	1,270	84.7	Japan
4	Southern Medical University	13	383	29.5	China
5	Shanghai Jiao Tong University	13	321	24.7	China
6	University of California	13	1,053	81	United States
7	China Medical University	12	200	16.7	China
8	Nankai University	11	395	35.9	China
9	Sun Yat-sen University	10	233	23.3	China
10	Chang Gung University	9	382	42.4	China

### 3.3 Analysis of authors

Using VOSviewer, a total of 3,915 scholars were identified as contributors to research on peripheral nerve injury and macrophages. Table 3 lists the top 10 most productive authors. Kiguchi, Norikazu and Kishioka, Shiroh ranked first jointly, with 13 publications each. Both authors received 1,117 total citations, with an average of 85.9 citations per publication, indicating substantial academic influence. Wang, Yu with 12 publications and Peng, Jiang with 11 publications followed closely and represented important contributors in this field. Notably, Zhang, Ji had the highest average number of citations per publication (118.1), reflecting the high quality and academic recognition of his research output. The author collaboration network map (Figure 4A) showed that several stable collaboration clusters have formed among core scholars. The Japanese research team represented by Kiguchi, Norikazu, Kishioka, Shiroh, and Kobayashi, Yuka formed the largest and most closely connected red cluster. The Chinese research team represented by Wang, Yu and Peng, Jiang constituted the blue collaboration cluster, while Chen, Yueh-Sheng and Yao, Chun-Hsu formed the green cluster. Overall, collaborations within the same country were relatively close, whereas cross-team collaborations between different clusters remained sparse. This suggests that further academic exchange among different research teams should be encouraged to promote collaborative development in this field.

**Table 3:** Top 10 most productive authors in research on macrophages in peripheral nerve injury.

Rank	Author	Publications	Citations	Average citations
1	Kiguchi, Norikazu	13	1,117	85.9
2	Kishioka, Shiroh	13	1,117	85.9
3	Wang, Yu	12	447	37.3
4	Peng, Jiang	11	470	42.7
5	Chen, Yueh-Sheng	10	162	16.2
6	Kobayashi, Yuka	9	947	105.2
7	Saika, Fumihiro	8	442	55.3
8	Yao, Chun-Hsu	8	161	20.1
9	Yi, Sheng	8	230	28.8
10	Zhang, Ji	8	945	118.1

**Figure 4:**
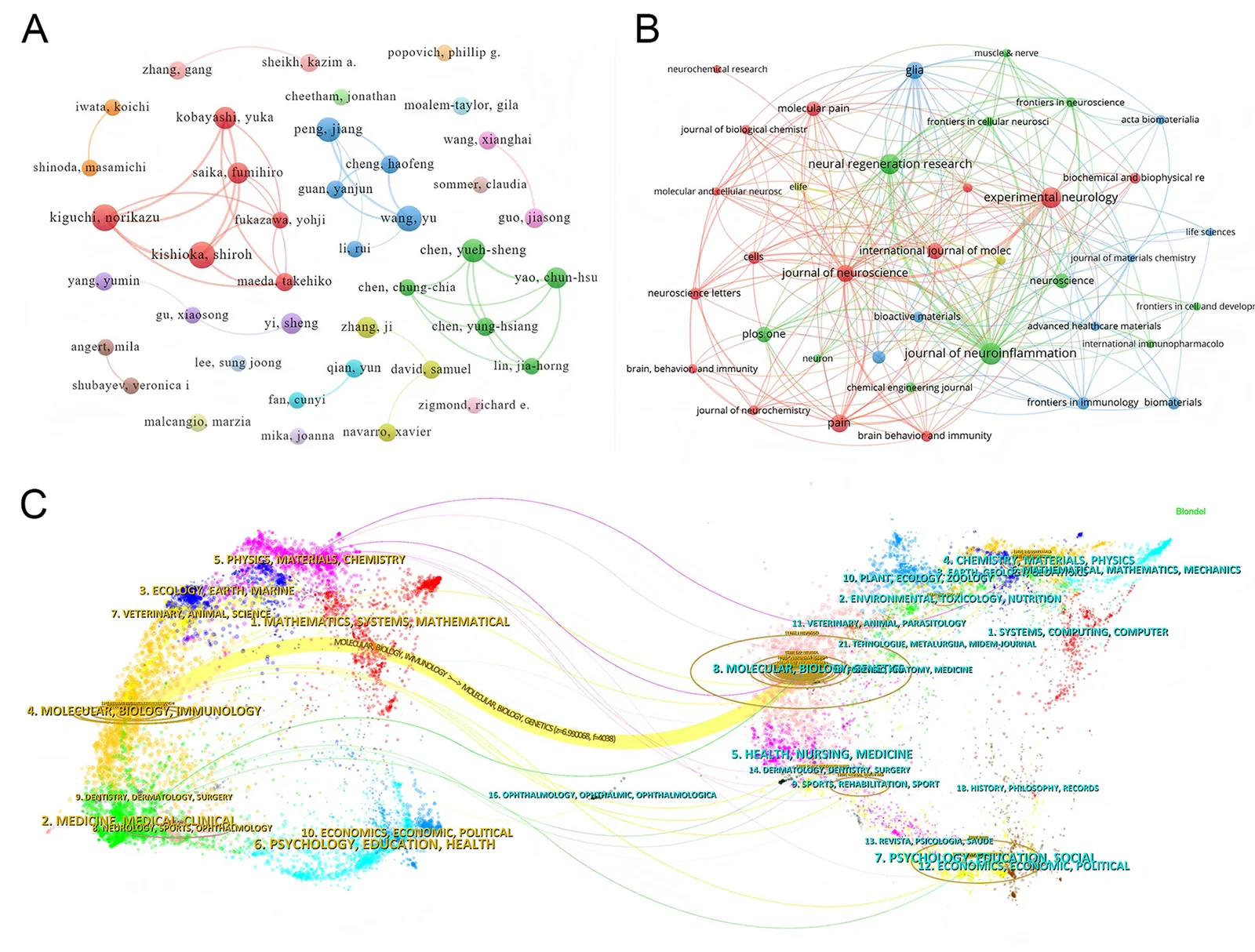
Visualized analysis of authors and journals in research on macrophages in peripheral nerve injury. **(A)** Network map of 40 core authors with more than five publications generated using VOSviewer. **(B)** Journal network map of 38 journals with more than four publications generated using VOSviewer. **(C)** Dual-map overlay of journals generated using CiteSpace.

### 3.4 Analysis of journals

A total of 317 journals published studies related to peripheral nerve injury and macrophages, among which 38 journals published four or more articles (Figure 4B), forming the core publication platforms in this field. Table 4 lists the top 10 journals by publication output. *Journal of Neuroinflammation* ranked first with 27 publications, followed by *Experimental Neurology* with 25 publications and *Neural Regeneration Research* with 24 publications. Together, these three journals accounted for nearly one-tenth of all publications in this field and served as the major publication outlets. Regarding journal quartiles and impact factors, seven of the top 10 journals were classified as JCR Q1 journals. The impact factors of *Journal of Neuroinflammation* (IF = 10.1), *Neural Regeneration Research* (IF = 6.7), and *Pain* (IF = 5.5) were relatively high in the fields of neuroscience and inflammation research, reflecting the academic authority and recognition of the core journals in this field.

**Table 4:** Top 10 most published journals in research on macrophages in peripheral nerve injury.

Rank	Journal	Publications	Citations	2024 JCR (IF)
1	Journal of Neuroinflammation	27	2,213	Q1 (10.1)
2	Experimental Neurology	25	1,189	Q1 (4.2)
3	Neural Regeneration Research	24	756	Q1 (6.7)
4	Pain	18	1,445	Q1 (5.5)
5	Glia	17	1,066	Q1 (5.1)
6	The Journal of Neuroscience	17	2,064	Q2 (4.0)
7	International Journal of Molecular Sciences	15	580	Q1 (4.9)
8	PLoS ONE	14	627	Q2 (2.6)
9	Molecular Pain	12	455	Q3 (2.8)
10	Neuroscience	12	438	Q3 (2.8)

The dual-map overlay of journals (Figure 4C) further revealed the interdisciplinary knowledge flow in this field. The cited journal clusters on the right side, representing the knowledge base, were mainly concentrated in “MOLECULAR, BIOLOGY, GENETICS,” whereas the citing journal clusters on the left side, representing current research frontiers, were mainly distributed in “MOLECULAR, BIOLOGY, IMMUNOLOGY.” The main citation paths, shown as yellow lines, extended from the right side to the left side, suggesting that research in this field is supported by foundational studies in molecular biology and genetics and is expanding toward frontier directions such as molecular immunology. This knowledge transition reflects the interdisciplinary characteristics of macrophage-related peripheral nerve injury research. The integration of molecular biology, genetics, and immunology suggests that studies in this field are increasingly drawing upon multiple biological disciplines to investigate the mechanisms underlying nerve injury and repair. In particular, the involvement of immunological concepts, including macrophage regulation, inflammatory responses, and cellular interactions, highlights the contribution of immune-related mechanisms to current research. Therefore, the dual-map overlay provides evidence of interdisciplinary knowledge exchange and indicates that macrophage-related peripheral nerve injury research is supported by molecular foundations while expanding toward immunological and mechanistic perspectives.

### 3.5 Analysis of keywords

Keywords are highly condensed representations of research content. Their frequency, co-occurrence relationships, and temporal evolution can intuitively reflect the core themes, research hotspots, and developmental trajectory of a field. In this study, VOSviewer and Bibliometrix were used to systematically analyze the keywords of publications related to peripheral nerve injury and macrophages from January 2006 to March 2026. The high-frequency keyword statistics (Table 5) showed that “peripheral nerve injury” ranked first, with an occurrence frequency of 434 and a total link strength of 7,914, indicating that it was the central theme throughout this field. “Macrophage” ranked second, with an occurrence frequency of 384 and a total link strength of 6,868, representing the core research object in this field. The frequent occurrence of keywords such as “Schwann cell,” “nerve regeneration,” and “sciatic nerve” indicated that Schwann cells and nerve regeneration are important research directions closely associated with macrophage-related studies. The keyword density map (Figure 5A) showed that the core keyword nodes were densely distributed, forming obvious core clusters around “peripheral nerve injury,” “macrophage,” “non-human,” and “animal,” reflecting the focused nature of the research topics.

**Table 5:** Top 10 most frequent keywords in research on macrophages in peripheral nerve injury.

Rank	Keyword	Occurrences	Total link strength
1	Peripheral nerve injury	434	7,914
2	Macrophage	384	6,868
3	Non-human	287	7,320
4	Animal	241	6,357
5	Article	232	6,319
6	Schwann cell	228	4,209
7	Controlled study	212	5,906
8	Animal experiment	207	5,828
9	Nerve regeneration	204	4,620
10	Sciatic nerve	204	4,371

**Figure 5:**
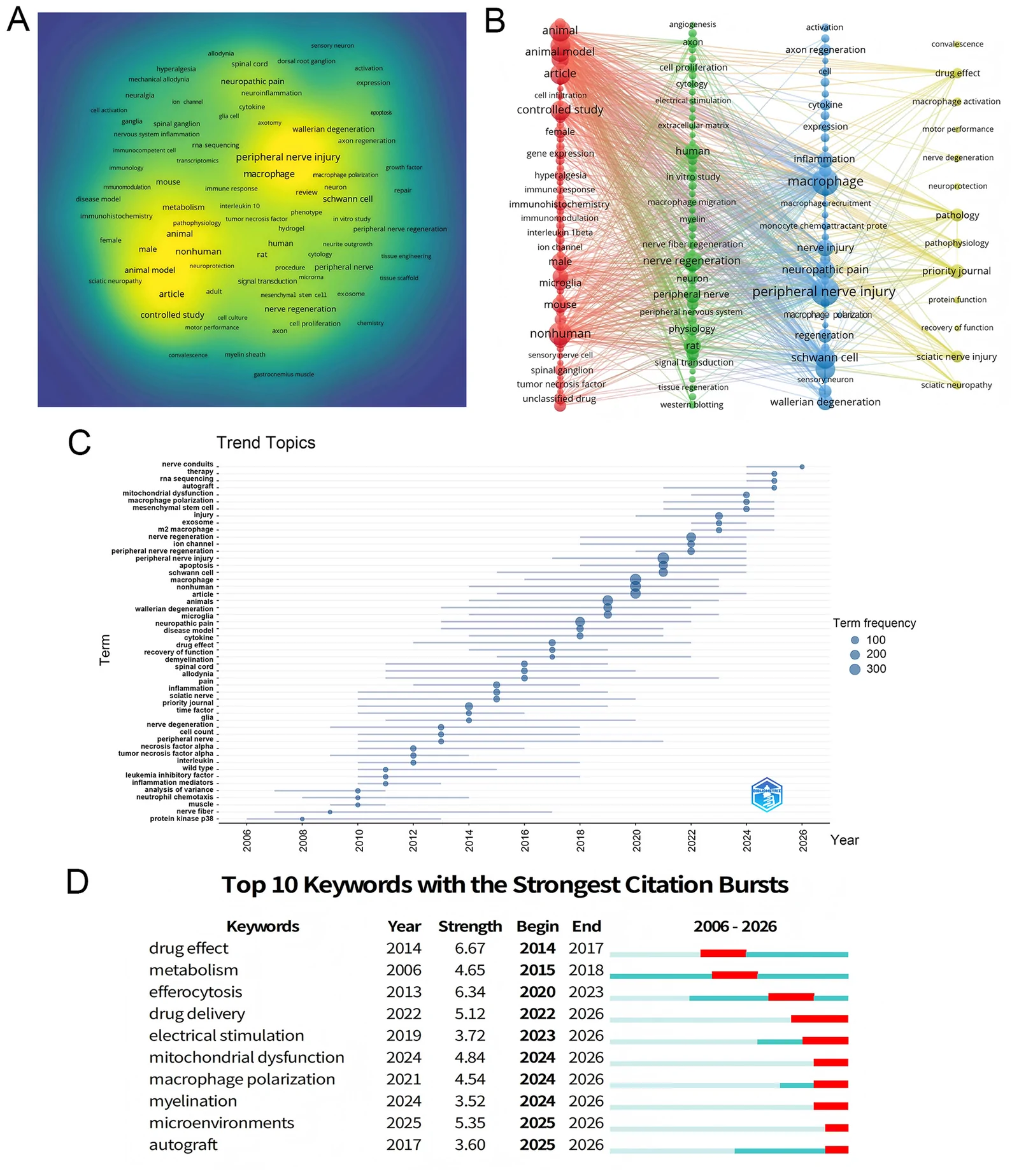
Visualized keyword analysis in research on macrophages in peripheral nerve injury. **(A)** Keyword density map generated using VOSviewer. **(B)** Keyword co-occurrence network generated using VOSviewer. Different colors represent different clusters, and the strength of the links reflects the degree of co-occurrence between keywords. **(C)** Thematic trend map generated using Bibliometrix. Node size is associated with keyword frequency, and horizontal lines indicate the time span of keywords and changes in research attention. **(D)** Top 10 keywords with the strongest citation bursts generated using CiteSpace. Higher burst strength indicates greater attention to a keyword during a specific period. Red bars indicate the duration during which the keyword remained in a high-burst state.

The keyword co-occurrence network map (Figure 5B) clearly identified four major research clusters in this field. The blue cluster was centered on “macrophage,” “peripheral nerve injury,” “neuropathic pain,” and “Wallerian degeneration,” and was associated with keywords such as “macrophage polarization,” “inflammation,” and “Schwann cell.” This cluster reflected the core pathological mechanism-related research directions, including macrophage polarization, neuropathic pain, Wallerian degeneration, and inflammatory responses. The green cluster was centered on “nerve regeneration” and included keywords such as “electrical stimulation,” “cell proliferation,” “axon,” “myelin,” “angiogenesis,” and “signal transduction.” This cluster focused on axonal growth, myelination, angiogenesis, and regulation of cell proliferation during nerve regeneration, representing an important research direction related to repair mechanisms. The red cluster was centered on “animal” and “controlled study” and was associated with keywords such as “immunomodulation” and “cell infiltration,” indicating that animal models are the main research carriers in this field and that immune-related processes, such as immunomodulation and cell infiltration, are important components of current research. The yellow cluster was centered on “sciatic nerve injury” and was associated with keywords such as “drug effect” and “neuroprotection,” reflecting studies on pharmacological interventions and neuroprotective strategies based on sciatic nerve injury models, and indicating a trend toward translational application.

The thematic evolution map (Figure 5C) revealed stage-specific shifts in research hotspots. In the early stage (2006–2015), studies mainly focused on basic pathological processes. Keywords such as “inflammation,” “interleukin,” and “tumor necrosis factor alpha” appeared frequently, suggesting that research during this period primarily explored inflammatory responses and related cytokine regulation. In the middle stage (2016–2020), the research focus shifted toward core pathological processes and cellular interactions. Keywords such as “neuropathic pain,” “Wallerian degeneration,” “macrophage,” and “Schwann cell” increased markedly in frequency, indicating that interactions between macrophages and Schwann cells became a major research hotspot during this period. In the recent stage (2021 to the present), the field has shown a dual trend of mechanistic deepening and interdisciplinary expansion. On the one hand, the rapidly increasing frequency of keywords such as “apoptosis,” “mitochondrial dysfunction,” and “ion channel” suggests that refined subcellular mechanisms, including apoptosis, mitochondrial dysfunction, and ion channel regulation, have become frontier research directions. On the other hand, keywords such as “nerve conduits,” “mesenchymal stem cell,” and “exosome” have recently emerged as important topics. These findings indicate that interdisciplinary repair strategies, including nerve conduits, mesenchymal stem cell therapy, and exosome delivery, are becoming important research directions. In addition, the appearance of “RNA sequencing” suggests that transcriptomic sequencing and other multi-omics technologies are emerging as important tools for mechanistic analysis. Collectively, the thematic evolution analysis demonstrates a progressive evolution of research focuses in peripheral nerve injury. The field has developed from early investigations of inflammatory responses and cytokine regulation toward broader exploration of macrophage-related mechanisms, cellular interactions, subcellular regulation, and emerging regenerative strategies. Recent keywords, including “mitochondrial dysfunction,” “ion channel,” “RNA sequencing,” “exosome,” and “nerve conduits,” reflect the increasing diversification and mechanistic refinement of research topics in this field.

CiteSpace was used to conduct keyword burst detection, and the top 10 keywords with the strongest burst strengths were identified (Figure 5D), providing a direct view of frontier dynamics in macrophage-related research in peripheral nerve injury at different stages. From 2014 to 2018, “drug effect” and “metabolism” entered periods of strong citation bursts, with burst strengths of 6.67 and 4.65, respectively, representing early core research hotspots and suggesting that studies during this period mainly focused on pharmacological interventions and metabolic regulation in basic pathological processes. From 2020 to 2023, “efferocytosis” emerged as a research frontier with a burst strength of 6.34, indicating that macrophage-mediated efferocytosis and its critical role in apoptotic cell clearance became a major focus during this period. Since 2022, the research frontier has shown a clear trend toward mechanistic refinement and application-oriented expansion. The continuing bursts of “drug delivery” from 2022 to 2026 with a strength of 5.12 and “electrical stimulation” from 2023 to 2026 with a strength of 3.72 indicate that drug delivery strategies and physical intervention methods have gradually become research hotspots. Meanwhile, the simultaneous bursts of “mitochondrial dysfunction,” “macrophage polarization,” and “microenvironments” from 2024 to 2026, with burst strengths of 4.84, 4.54, and 5.35, respectively, suggest that refined mechanisms such as macrophage polarization regulation, mitochondrial dysfunction, and injury microenvironment remodeling have become among the most active research directions in this field.

### 3.6 Analysis of citations

Citation analysis is an important method for identifying highly influential publications and tracing the evolution of knowledge within a field. In this study, VOSviewer and CiteSpace were used to perform citation analysis of the 694 included publications, with the aim of identifying core literature and developmental trajectories in macrophage-related research in peripheral nerve injury. The citation network generated by VOSviewer is shown in Figure 6A, which visually presents the citation relationships among the included publications. Table 6 lists the top 10 most frequently cited articles. Most of these highly cited publications were published in high-level JCR Q1 journals, including authoritative platforms such as *Nature Medicine, Nature Neuroscience*, and *Journal of Neuroinflammation*. Their topics mainly focused on Wallerian degeneration, neuroinflammation, neuropathic pain, and macrophage functional regulation. Among them, the review by Gaudet et al. (2011), published in *Journal of Neuroinflammation*, ranked first with 759 citations. This review systematically described inflammatory events during Wallerian degeneration after peripheral nerve injury and laid an important theoretical foundation for subsequent studies. The study by Ren and Dubner (2010), published in *Nature Medicine*, ranked second with 713 citations and revealed interactions between the immune system and the nervous system in pain regulation, providing key evidence for immune mechanisms underlying neuropathic pain. Together, these highly cited publications indicate that Wallerian degeneration, neuroinflammation, and macrophage-mediated immune regulation have long been core research directions in this field.

**Figure 6:**
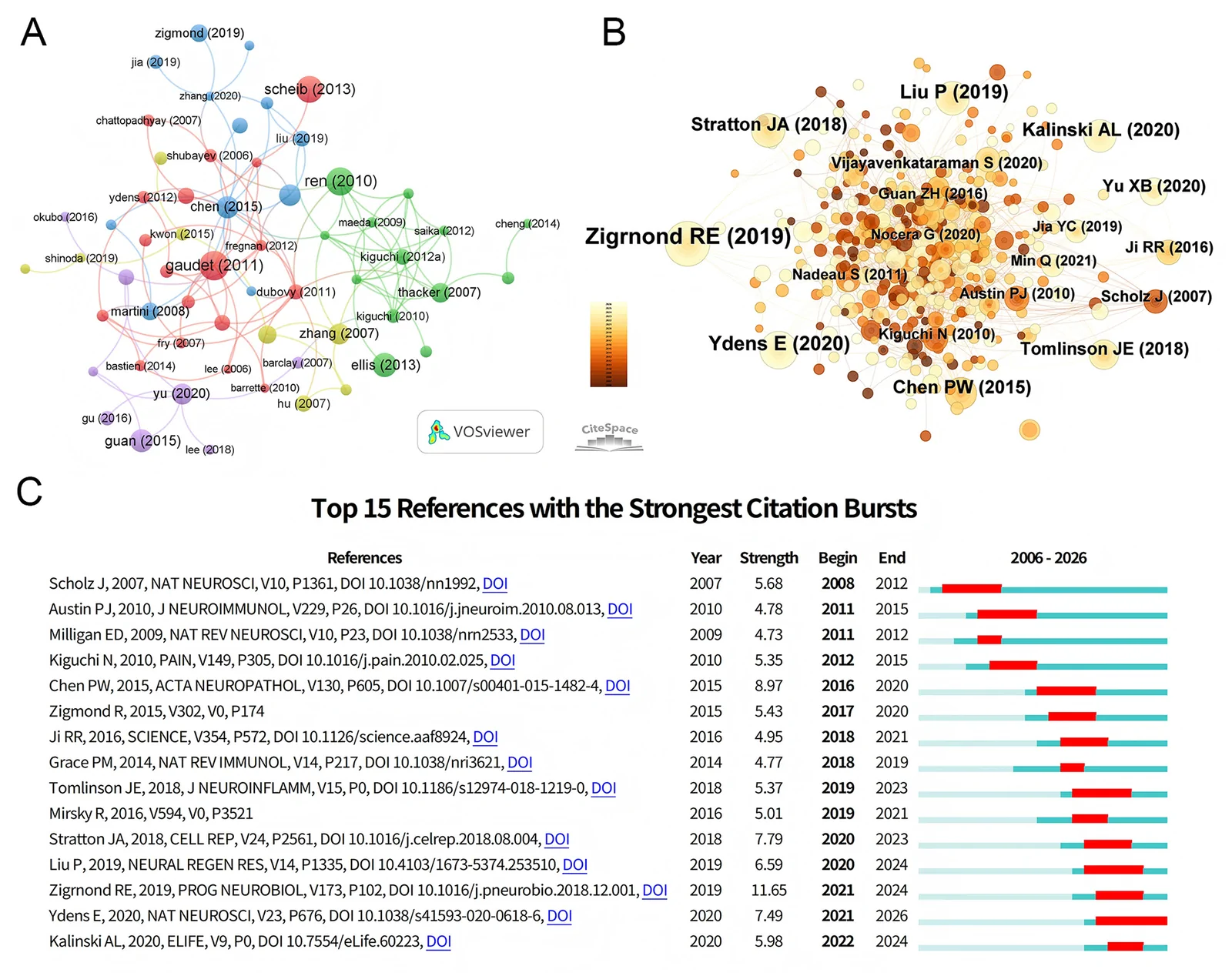
Visualized citation analysis in research on macrophages in peripheral nerve injury. **(A)** Citation network map generated using VOSviewer. **(B)** Co-cited reference network generated using CiteSpace. **(C)** Top 15 co-cited references with the strongest citation bursts generated using CiteSpace. Burst intensity reflects the degree of attention received by a reference during a specific period, and the start and end times of the burst indicate the duration during which the reference remained highly influential.

**Table 6:** Top 10 most cited papers in research on macrophages in peripheral nerve injury.

Rank	References	Paper	Citations	Journal	2024 JCR (IF)
1	Gaudet et al., 2011	Wallerian degeneration: gaining perspective on inflammatory events after peripheral nerve injury	759	Journal of Neuroinflammation	Q1 (10.1)
2	Ren and Dubner, 2010	Interactions between the immune and nervous systems in pain	713	Nature Medicine	Q1 (50.0)
3	Scheib and Höke, 2013	Advances in peripheral nerve regeneration	641	Nature Reviews Neurology	Q1 (33.1)
4	Ellis and Bennett, 2013	Neuroinflammation and the generation of neuropathic pain	520	British Journal of Anesthesia	Q1 (9.2)
5	Guan et al., 2016	Injured sensory neuron-derived SCF1 induces microglial proliferation and DAP12-dependent pain	467	Nature Neuroscience	Q1 (20.0)
6	Chen et al., 2015	Role of macrophages in Wallerian degeneration and axonal regeneration after peripheral nerve injury	441	Acta Neuropathologica	Q1 (9.3)
7	Rotshenker, 2011	Wallerian degeneration: the innate-immune response to traumatic nerve injury	422	Journal of Neuroinflammation	Q1 (10.1)
8	Yu et al., 2020	Dorsal root ganglion macrophages contribute to both the initiation and persistence of neuropathic pain	390	Nature Communications	Q1 (15.7)
9	Zhang et al., 2007	Expression of CCR2 in both resident and bone marrow-derived microglia plays a critical role in neuropathic pain	383	The Journal of Neuroscience	Q2 (4.0)
10	Thacker et al., 2007	Pathophysiology of peripheral neuropathic pain: immune cells and molecules	342	Anesthesia & Analgesia	Q1 (3.8)

The co-cited reference network map (Figure 6B) reflected the knowledge association structure among references. Node size represented the co-citation frequency of each reference, line thickness indicated the strength of associations between references, and different colors represented different publication years. Table 7 lists the top 10 co-cited references. The review by Zigmond and Echevarria (2019) ranked first with 37 co-citations and systematically summarized macrophage biology after peripheral nervous system injury. The study by Ydens et al. (2020) ranked second with 25 co-citations and used transcriptomic analysis to reveal the distinct localization and functional responses of macrophage subsets in peripheral nerve injury, providing an important reference for research on macrophage heterogeneity. These core references occupied hub positions in the co-citation network and served as key supporting nodes in the knowledge system of this field.

**Table 7:** Top 10 co-cited references in research on macrophages in peripheral nerve injury.

Rank	First author (year)	Reference	Citations	Journal
1	Zigmond and Echevarria, 2019	Macrophage biology in the peripheral nervous system after injury	37	Progress In Neurobiology
2	Ydens et al., 2020	Profiling peripheral nerve macrophages reveals two macrophage subsets with distinct localization, transcriptome and response to injury	25	Nature Neuroscience
3	Liu et al., 2019	Role of macrophages in peripheral nerve injury and repair	21	Neural Regeneration Research
4	Chen et al., 2015	Role of macrophages in Wallerian degeneration and axonal regeneration after peripheral nerve injury	19	Acta Neuropathol
5	Kalinski et al., 2020	Analysis of the immune response to sciatic nerve injury identifies efferocytosis as a key mechanism of nerve debridement	19	Elife
6	Stratton et al., 2018	Macrophages Regulate Schwann Cell Maturation after Nerve Injury	19	Cell Reports
7	Tomlinson et al., 2018	Temporal changes in macrophage phenotype after peripheral nerve injury	15	Journal Of Neuroinflammation
8	Yu et al., 2020	Dorsal root ganglion macrophages contribute to both the initiation and persistence of neuropathic pain	14	Nature Communications
9	Scholz and Woolf, 2007	The neuropathic pain triad: neurons, immune cells and glia	11	Nature Neuroscience
10	Vijayavenkataraman, 2020	Nerve guide conduits for peripheral nerve injury repair: a review on design, materials and fabrication methods	11	Acta Biomaterialia

Citation burst analysis (Figure 6C) further revealed frontier dynamics and the evolution of knowledge in this field. In the early stage (2008–2015), publications by Scholz and Woolf (2007) and Kiguchi et al. (2010) successively entered periods of strong citation bursts, laying the foundation for research on “neuropathic pain–immune cell interactions.” In the middle stage (2016–2020), the study by Chen et al. (2015) became a core hotspot with a burst strength of 8.97. This work clarified the role of macrophages in Wallerian degeneration and axonal regeneration, thereby promoting a more systematic understanding of macrophage function in this field. In the recent stage (2021 to the present), publications by Zigmond and Echevarria (2019) (burst strength, 11.65), Ydens et al. (2020) (burst strength, 7.49), and Kalinski et al. (2020) (burst strength, 5.98) have remained in periods of strong citation bursts. Among them, the review by Zigmond and the macrophage subset study by Ydens have become the most prominent frontier publications. These findings indicate that research in this field is shifting from macroscopic inflammatory regulation toward more refined directions, such as macrophage heterogeneity and immune functional remodeling, while also reflecting the extension of macrophage-related basic research toward clinical translational applications.

## 4 Discussion

### 4.1 Summary of basic information

In this study, bibliometric methods were used to systematically analyze 694 publications related to peripheral nerve injury and macrophages from January 2006 to March 2026. Overall, the annual number of publications showed a steady upward trend, entering a phase of rapid growth after 2017 and reaching a peak of 76 publications in 2025. The high goodness of fit of the exponential model for cumulative publications indicated that this field has developed continuously and steadily, reflecting increasing research interest. A total of 50 countries contributed to this field. China ranked first with 263 publications, followed by the United States and Japan, forming the core research contributors. The United States showed a stronger performance in both total citations and average citations per publication, suggesting its leading role in research quality, which may be attributed to sufficient research funding and advanced technological platforms. International collaboration showed a dual-core pattern centered on China and the United States, with the closest collaborative links observed between these two countries. However, some countries showed relatively low participation. Among the top 10 institutions by publication output, eight were from China, with Nantong University ranking first globally. Although domestic institutional collaboration was relatively close, cross-national collaboration remained limited, indicating the need to further strengthen international cooperation.

A total of 3,915 scholars contributed to this field. Kiguchi, Norikazu and Kishioka, Shiroh ranked first jointly in publication output. Research teams from China and Japan formed several stable collaboration clusters; however, cross-team and cross-national collaborations remained relatively weak. Most core author clusters were dominated by domestic collaboration, while international linkage was insufficient, which may limit the rapid sharing of frontier findings and innovative breakthroughs. The included publications were distributed across 317 journals, with *Journal of Neuroinflammation, Experimental Neurology*, and *Neural Regeneration Research* ranking as the top three journals by publication output. Most of the top 10 journals were JCR Q1 journals. The dual-map overlay of journals showed that research in this field is based mainly on molecular biology and genetics and is gradually extending toward molecular immunology and related directions.

### 4.2 Research hotspots and trends

#### 4.2.1 Macrophage polarization, wallerian degeneration, and neuropathic pain mechanisms

The keyword co-occurrence, density, and thematic evolution analyses collectively demonstrated that macrophage-related research themes represent a core knowledge structure in the field of peripheral nerve injury. In the keyword density map, prominent keywords including “macrophage polarization,” “Wallerian degeneration,” and “neuropathic pain” showed dense distribution, reflecting their sustained research attention and close association within this field. Thematic evolution analysis showed that during the middle stage of research development (2016–2020), keywords such as “neuropathic pain,” “Wallerian degeneration,” and “macrophage” exhibited marked increases in frequency, indicating a transition of research focus from general inflammatory responses toward macrophage-associated pathological mechanisms and functional regulation. Furthermore, the keyword co-occurrence network identified a major cluster centered on “macrophage,” “peripheral nerve injury,” “neuropathic pain,” and “Wallerian degeneration,” with closely related terms including “macrophage polarization” and “inflammation.” These network characteristics suggest that macrophage-related inflammatory regulation, Wallerian degeneration, and neuropathic pain constitute an interconnected mechanistic framework rather than independent research topics.

After peripheral nerve injury, Wallerian degeneration occurs in the distal nerve segment and involves axonal fragmentation, myelin breakdown, inflammatory cell recruitment, and debris clearance (Yamamoto et al., 2022). The central position of Wallerian degeneration in the citation network reflects its role as a foundational concept for understanding macrophage involvement after nerve injury. Previous studies have demonstrated that Wallerian degeneration is not merely a passive degenerative process but an actively regulated event that creates conditions for subsequent repair. Macrophages contribute to this process by clearing axonal and myelin debris and regulating inflammatory responses (Zigmond and Echevarria, 2019). Gaudet et al. (2011), the most highly cited publication in this field, provided an important conceptual framework for inflammatory events during Wallerian degeneration (Gaudet et al., 2011), whereas Chen et al. (2015) further clarified macrophage functions during Wallerian degeneration and axonal regeneration (Chen et al., 2015). The high citation impact and central position of these studies within the citation network indicate that they represent key knowledge nodes that shaped subsequent investigations of macrophage-mediated nerve injury mechanisms.

Macrophage polarization is an important mechanism explaining the dual roles of macrophages after peripheral nerve injury. In the early stage after injury, M1 macrophages are activated by signals such as LPS, IFN-γ, and TNF-α, exhibiting increased expression of pro-inflammatory markers including CD86 and iNOS and releasing inflammatory mediators such as TNF-α, IL-1β, and IL-6. These responses facilitate the clearance of necrotic tissue and myelin debris; however, excessive or prolonged M1 activation may aggravate local inflammatory responses, enhance sensory neuron excitability, and contribute to neuropathic pain development. As the injury progresses toward the repair phase, M2 macrophages are induced by signals including IL-4, IL-13, IL-10, and TGF-β, and promote inflammation resolution and tissue restoration through the secretion of anti-inflammatory and trophic factors (Liu et al., 2019; Chen et al., 2023; Zhao et al., 2023). Therefore, the timely transition from pro-inflammatory to repair-associated macrophage states and the dynamic balance of macrophage polarization are important determinants of inflammatory outcomes, Wallerian degeneration progression, nerve repair efficiency, and pain prognosis.

Neuropathic pain represents another important research axis within the macrophage-centered network and reflects the clinical consequences of macrophage-mediated pathological regulation after peripheral nerve injury. Following nerve injury, macrophages accumulate not only at the lesion site but also in sensory-related regions such as the dorsal root ganglia. By releasing inflammatory mediators, including TNF-α, IL-1β, IL-6, and CCL2, macrophages influence sensory neuron excitability and contribute to peripheral sensitization and abnormal pain responses (Silva et al., 2021; Zeboudj et al., 2023). Yu et al. (2020) demonstrated that dorsal root ganglion macrophages participate in the initiation and maintenance of mechanical hypersensitivity after nerve injury, suggesting that the “macrophage–inflammatory factor–sensory neuron excitability” axis represents an important mechanism underlying neuropathic pain. These findings further explain why neuropathic pain remains a persistent core topic within the keyword network, as macrophages serve as a functional link between nerve injury and abnormal sensory processing. Therefore, macrophage polarization, Wallerian degeneration, and neuropathic pain are not isolated research topics; rather, they constitute a continuous pathological process involving injury responses, macrophage functional regulation, and pain development. In the future, how to preserve the beneficial clearance and repair functions of macrophages while preventing excessive activation associated with pathological pain will remain an important direction for basic research and translational intervention.

#### 4.2.2 Macrophage–Schwann cell interactions and regulation of nerve regeneration

Keyword analysis revealed that “macrophage” and “Schwann cell” were among the most frequently occurring keywords in this field, ranking second and sixth, respectively, indicating their central roles in macrophage-related peripheral nerve injury research. In the keyword co-occurrence network, the blue cluster was mainly centered on “macrophage” and “Schwann cell,” whereas the green cluster was centered on “nerve regeneration” and included closely related keywords such as “axon” and “myelin.” These clustering patterns demonstrate that macrophage–Schwann cell interactions are closely associated with regenerative processes, including axonal growth and myelin repair. Furthermore, thematic evolution analysis showed that during the middle stage of research development (2016–2020), research attention gradually shifted toward cellular interactions, with keywords such as “macrophage” and “Schwann cell” showing marked increases in frequency. These findings suggest that macrophage–Schwann cell interactions have become a key research direction linking injury responses with nerve regeneration.

Following peripheral nerve injury, Schwann cells rapidly transition from a mature myelinating phenotype to a repair-associated state, while macrophages are recruited to the injured nerve and participate in debris clearance and regeneration regulation. Schwann cells provide a regenerative scaffold and produce signals that support axonal extension, whereas macrophages contribute to regeneration by promoting myelin debris removal, angiogenesis, Schwann cell maturation, and remyelination (Huang et al., 2024; Jessen and Arthur-Farraj, 2019; Wu et al., 2023). Cattin et al. (2015) demonstrated that macrophage-mediated angiogenesis provides vascular guidance structures that facilitate Schwann cell migration and axonal regeneration, revealing a coordinated process involving macrophages, blood vessels, Schwann cells, and regenerating axons. In addition, Stratton et al. (2018), an important co-cited reference in this field, showed that macrophages regulate Schwann cell maturation after nerve injury, supporting the concept that macrophages participate throughout multiple stages of nerve repair rather than only during the initial clearance phase. Therefore, macrophage–Schwann cell interactions represent a dynamic regulatory axis in peripheral nerve regeneration. Future studies focusing on the molecular communication, spatial organization, and functional coordination between these two cell types may provide new strategies for enhancing axonal regeneration, remyelination, and functional recovery.

#### 4.2.3 Immune cell infiltration and inflammatory microenvironment remodeling

After peripheral nerve injury, local tissues rapidly form an inflammatory microenvironment composed of immune cells, Schwann cells, fibroblasts, vascular endothelial cells, chemokines, inflammatory factors, and extracellular matrix components. This microenvironment does not merely represent an injury response; rather, it also contributes to tissue clearance, inflammatory regulation, and regenerative support. Consistent with the keyword co-occurrence analysis, immune-related processes represented by “immunomodulation” and “cell infiltration” formed an important research cluster, highlighting that immune regulation and cellular recruitment are essential components of peripheral nerve injury studies. Furthermore, keyword burst analysis identified “microenvironments” as an emerging topic in 2025–2026, suggesting that recent research interest has shifted toward understanding the dynamic remodeling of the injury microenvironment. The keyword co-occurrence and burst analyses in this study showed that “immunomodulation,” “cell infiltration,” and “microenvironments” appeared continuously, suggesting that research in this field has gradually shifted from a simple focus on inflammatory factor expression toward immune cell infiltration patterns, cellular behavior regulation, and overall remodeling of the injury microenvironment. Unlike the action of a single cell type or a single factor, the repair outcome after peripheral nerve injury depends more on the dynamic balance among multiple cell types and inflammatory mediators. In particular, the timing, intensity, and spatial distribution of immune cell infiltration play important roles in determining whether the inflammatory response can smoothly transition from the acute clearance phase to the repair phase.

Immune cell infiltration is an early key event in the formation of the injury microenvironment. After nerve injury, the permeability of the blood–nerve barrier increases, and local cells release chemokines such as CCL2 and CCL3, thereby promoting the recruitment of circulating monocytes and other immune cells to the injured region (Wang et al., 2025). Among these signals, the CCL2/CCR2 axis is considered one of the important pathways regulating macrophage recruitment and is associated with angiogenesis, cell repopulation, and nerve regeneration (Pan et al., 2020). However, some studies have suggested that CCL2 is not the sole determinant of macrophage accumulation and regenerative repair under all injury conditions, indicating that immune cell recruitment is jointly regulated by multiple chemotactic signals and the local microenvironment (Talsma et al., 2022). Therefore, the emerging concept of inflammatory microenvironment remodeling emphasizes not only the recruitment of immune cells but also the coordinated regulation of their timing, distribution, and functional effects within injured tissues. Accordingly, future precise interventions targeting chemokine pathways, immune cell infiltration patterns, and inflammatory microenvironment remodeling may become an important direction for promoting peripheral nerve repair.

#### 4.2.4 Subcellular injury and neural excitability regulation: apoptosis and efferocytosis, mitochondrial dysfunction, and ion channels

Keyword burst analysis showed that “efferocytosis” exhibited a strong burst from 2020 to 2023, indicating that macrophage-mediated apoptotic cell clearance has become an emerging research focus during this period. After peripheral nerve injury, local Schwann cells, immune cells, and sensory neurons may undergo varying degrees of apoptosis or secondary cell death. If apoptotic cells are not cleared in a timely manner, they may further release damage-associated molecular patterns, prolong inflammatory responses, and disrupt the repair microenvironment (Gu et al., 2024). Efferocytosis is an important mechanism by which macrophages clear apoptotic cells and promote inflammation resolution. Unlike the simple phagocytosis of necrotic tissue or myelin debris, efferocytosis emphasizes macrophage-mediated recognition, engulfment, and immune homeostatic regulation of apoptotic cells. The citation burst analysis identified Kalinski et al. (2020) as an influential reference, highlighting the important role of this study in promoting the recognition of efferocytosis as a key regulatory mechanism after nerve injury. Using single-cell RNA sequencing, Kalinski et al. (2020) identified multiple macrophage subsets in injured sciatic nerves, some of which cleared apoptotic leukocytes through efferocytosis and promoted the formation of an anti-inflammatory microenvironment. These findings indicate that efferocytosis is not merely a process of “tissue cleanup,” but an important link connecting cell death, inflammation resolution, and immune homeostasis reconstruction. This conceptual shift from viewing macrophages as simple debris-clearing cells toward recognizing efferocytosis as an active regulatory process linking apoptotic cell clearance with tissue homeostasis may explain why efferocytosis emerged as a prominent research hotspot in recent years.

Thematic evolution analysis showed that “mitochondrial dysfunction” has rapidly increased in recent years, and keyword burst analysis further demonstrated a continuous burst from 2024 to 2026 (burst strength = 4.84), suggesting that mitochondrial regulation has become a newly emerging frontier in peripheral nerve injury research. Mitochondria not only provide energy for axonal transport, ion pump activity, and nerve impulse conduction, but also participate in calcium homeostasis maintenance, reactive oxygen species production, and apoptosis regulation. After injury, decreased mitochondrial membrane potential, reduced ATP production, enhanced oxidative stress, and abnormal mitochondrial dynamics may impair neuronal and Schwann cell function, thereby further affecting axonal regeneration and myelin repair (Wang et al., 2021). Related reviews have indicated that mitochondrial injury may contribute to the development and progression of neuropathic pain and peripheral neuropathy through energy depletion, oxidative stress, calcium homeostasis disruption, and abnormal mitophagy. In addition, mitochondrial function in Schwann cells is also essential for maintaining axonal survival and peripheral nerve function (Li Y. et al., 2025). Therefore, mitochondrial dysfunction may serve as an important intermediate link connecting subcellular injury, metabolic imbalance, impaired nerve repair, and neuropathic pain development. The increasing recognition of mitochondria as dynamic regulators of energy metabolism, oxidative stress, calcium homeostasis, and cellular survival, rather than merely passive energy-producing organelles, may explain why mitochondrial dysfunction has recently emerged as a frontier topic in peripheral nerve injury research.

Consistent with the thematic evolution analysis, “ion channel” has shown increasing activity in recent years, reflecting growing interest in the regulation of neural excitability after peripheral nerve injury. Ion channel abnormalities mainly reflect changes in sensory neuron excitability after peripheral nerve injury. After injury, local inflammatory factors, chemokines, and macrophage-derived signals can affect the electrophysiological state of dorsal root ganglion sensory neurons, leading to altered expression or function of voltage-gated sodium channels, TRP channels, potassium channels, and calcium channels. These changes reduce the neuronal excitability threshold and induce abnormal discharges and sensory sensitization (Alles and Smith, 2021). Taking TRPV1 as an example, tissue injury or inflammatory conditions can promote TRPV1 sensitization and enhance pain signal transmission in primary afferent neurons (Gao et al., 2024). Moreover, studies on the IL-23/IL-17A/TRPV1 axis further suggest that macrophage-derived inflammatory signals can affect sensory neuron excitability by regulating TRP channels, thereby contributing to mechanical pain and pain sensitization (Luo et al., 2021). In addition, inflammatory signals such as IL-1β can upregulate Nav1.7 expression in sensory neurons, further supporting the important role of the “immune cell–inflammatory signal–ion channel–neural excitability” axis in the formation of abnormal sensory responses after peripheral nerve injury (Zhang et al., 2018). This transition from describing inflammatory pain mediators toward understanding precise regulation of neuronal excitability also contributes to the increasing attention received by ion channel-related mechanisms in recent years.

Taken together, apoptosis and efferocytosis, mitochondrial dysfunction, and ion channel abnormalities jointly constitute an important mechanistic network of subcellular injury and altered neural excitability after peripheral nerve injury. Compared with traditional studies focusing mainly on inflammatory cell accumulation and cytokine expression, current research increasingly emphasizes the roles of apoptotic cell clearance efficiency, mitochondrial homeostasis maintenance, and ion channel functional regulation in nerve repair. In the future, studies aimed at enhancing macrophage efferocytosis, protecting mitochondrial function, suppressing oxidative stress, and precisely regulating ion channels may provide new interventional targets for improving the injury microenvironment, reducing abnormal neural excitability, and promoting functional recovery.

#### 4.2.5 Tissue engineering and biotherapeutic strategies: nerve conduits, mesenchymal stem cells, exosomes, and drug delivery

The keyword trend and burst analyses indicate that tissue engineering and biotherapeutic strategies have become increasingly important directions in peripheral nerve injury research. Thematic evolution analysis showed that, during the recent stage (2021 to the present), keywords including “nerve conduits,” “mesenchymal stem cell,” and “exosome” have emerged as rapidly developing topics, reflecting the increasing integration of biomaterials, cell-based therapy, and extracellular vesicle-based approaches. In addition, keyword burst analysis demonstrated that “drug delivery” (2022–2026, burst strength = 5.12) and “electrical stimulation” (2023–2026, burst strength = 3.72) remained active emerging topics, indicating that controlled therapeutic delivery and physical modulation strategies are becoming important components of peripheral nerve repair. In contrast to the traditional concept of simply bridging nerve defects, current designs of nerve conduits and biomaterials increasingly emphasize integrated functions involving “structural support, immunomodulation, and regeneration induction.” This is achieved by regulating local immune responses and the regenerative microenvironment through material pore size, degradation rate, surface morphology, electrical conductivity, and bioactive molecule release (Vijayavenkataraman, 2020; Wan et al., 2023). Recent reviews on immunomodulatory nerve conduits have also indicated that natural and synthetic materials, such as collagen, chitosan, silk fibroin, PLGA, PLA, and PCL, can influence macrophage responses through different mechanisms and thereby participate in peripheral nerve repair (Zhang et al., 2025).

In tissue engineering strategies, nerve conduits not only provide physical channels for regenerating axons, but also serve as delivery platforms for drugs, growth factors, cells, or exosomes. Functionalized conduits can alleviate excessive inflammatory responses and promote angiogenesis, Schwann cell migration, and axonal extension by enabling the sustained release of anti-inflammatory drugs, neurotrophic factors, nucleic acid molecules, or immunomodulatory factors (Manoukian et al., 2020). The increasing attention toward nerve conduits in thematic evolution analysis reflects a conceptual shift from passive structural replacement toward active regulation of the regenerative process. This transition may be attributed to the recognition that successful peripheral nerve repair requires not only physical guidance for axonal growth but also precise regulation of the local regenerative environment, including immune responses, vascular support, and Schwann cell-mediated repair processes. Some novel conductive or aligned nerve conduits can also mimic the electrophysiological properties and spatial arrangement of neural tissue, providing a microenvironment that more closely resembles physiological conditions for directed axonal growth. For example, a 2024 study showed that electrically aligned polyurethane nerve guidance conduits could regulate macrophage responses and promote immunoregulatory peripheral nerve regeneration, indicating that the physical properties of biomaterials themselves may also be important factors in regulating the immune microenvironment (Sun et al., 2024). Therefore, the focus of nerve conduit research is shifting from whether a conduit can bridge a defect to whether it can actively remodel an immune microenvironment favorable for regeneration.

Mesenchymal stem cells, exosomes, and drug delivery represent another important translational direction in biotherapeutic strategies. Mesenchymal stem cells possess paracrine, immunomodulatory, and pro-regenerative effects and can regulate inflammatory responses and tissue repair by releasing cytokines, growth factors, and extracellular vesicles (Li X. et al., 2022). As an important type of extracellular vesicle, exosomes are relatively stable, have low immunogenicity, and are amenable to engineering modification; therefore, they have been widely applied in peripheral nerve injury repair in recent years (Tan et al., 2024). The recent emergence of “exosome” and “mesenchymal stem cell” in thematic evolution analysis reflects the growing interest in cell-free and cell-based regenerative approaches. This trend reflects the limitations of conventional single-material repair strategies and the increasing demand for biologically active therapies capable of modulating inflammation, enhancing regeneration, and improving functional recovery. Related reviews have suggested that combining mesenchymal stem cells or their exosomes with nerve conduits is an important developmental direction for the treatment of peripheral nerve injury (Li M. et al., 2025). Studies have also shown that mesenchymal stem cell-derived exosomes can promote facial nerve injury recovery by regulating macrophage responses, and that exosomes derived from LPS-preconditioned bone marrow mesenchymal stem cells can promote peripheral nerve regeneration through the TSG-6/NF-κB/NLRP3-related pathway (Xue et al., 2024). Therefore, future tissue engineering and biotherapeutic strategies may no longer be limited to a single material or a single cell therapy. Instead, they may develop toward composite approaches integrating nerve conduits, immunomodulatory materials, exosomes or mesenchymal stem cells, and precise drug delivery, thereby achieving coordinated regulation of the injury microenvironment, macrophage function, and nerve regeneration.

#### 4.2.6 Transcriptomics and single-cell RNA sequencing drive the analysis of macrophage heterogeneity

The thematic trend analysis showed that keywords such as “RNA sequencing” have appeared in recent years, suggesting that macrophage-related research in peripheral nerve injury is shifting from traditional whole-tissue-level analysis toward refined mechanistic analysis represented by transcriptomics and single-cell RNA sequencing. This trend indicates that recent studies are increasingly focusing on resolving cellular heterogeneity and functional states that cannot be fully captured by conventional approaches. Traditional bulk RNA sequencing can reflect overall gene expression changes in injured nerve tissue. However, the local tissue composition after peripheral nerve injury is complex and includes macrophages, Schwann cells, fibroblasts, vascular endothelial cells, and other immune cells. As a result, bulk sequencing can easily mask differences among distinct cell types and cell subsets. Therefore, transcriptomic changes identified through bulk sequencing may not accurately represent the specific contributions of individual cell populations during nerve injury progression and repair. By contrast, single-cell RNA sequencing can identify different cell populations, transcriptional states, and cell–cell communication networks at cellular resolution, enabling researchers to move beyond the question of whether macrophages increase to more precise questions, such as which macrophage subsets function at which time points and locations, and what roles they play (Li and Wang, 2021). Therefore, the application of transcriptomics and single-cell RNA sequencing provides important technical support for dissecting the complex immune microenvironment, cellular heterogeneity, and cell interaction networks after peripheral nerve injury.

Macrophage heterogeneity is one of the core issues advanced by transcriptomics and single-cell RNA sequencing in this field. The citation analysis further highlights the importance of this research direction. Ydens et al. (2020) ranked second among highly co-cited references with 25 co-citations, while Zigmond and Echevarria (2019), Ydens et al. (2020), and Kalinski et al. (2020) showed sustained citation bursts from 2021 onward, with burst strengths of 11.65, 7.49, and 5.98, respectively. These findings indicate that macrophage heterogeneity and functional remodeling have become major knowledge frontiers in this field. This shift in research interest is driven by the increasing recognition that macrophages exhibit diverse origins, transcriptional programs, and functional states during different stages of peripheral nerve injury, making a single M1/M2 classification insufficient to explain their complex roles in injury progression and repair. Ydens et al. identified macrophage subsets in peripheral nerves with distinct localization, transcriptional characteristics, and injury responses through transcriptomic analysis (Ydens et al., 2020). Kalinski et al. (2020) further identified multiple macrophage subsets in injured sciatic nerves using single-cell RNA sequencing and found that some macrophages participated in the phagocytosis of apoptotic leukocytes and promoted the formation of an anti-inflammatory microenvironment. These studies suggest that although the traditional M1/M2 classification is useful for summarizing the pro-inflammatory and reparative functions of macrophages, it is insufficient to fully explain the complexity of macrophages *in vivo* in terms of their origin, spatial distribution, transcriptional states, and functional lineages. More importantly, single-cell RNA sequencing can reveal ligand–receptor interactions between macrophages and other cell types, including Schwann cells, fibroblasts, and vascular endothelial cells. This approach may help identify key regulatory pathways and potential therapeutic windows at different stages of injury. In the future, the integration of single-cell RNA sequencing, spatial transcriptomics, multi-omics approaches, and functional validation experiments is expected to establish a comprehensive mechanistic framework linking “cell subsets, spatial localization, molecular pathways, and regenerative outcomes” after peripheral nerve injury, and to provide a new theoretical basis for macrophage-targeted regulation and precise regenerative therapy.

### 4.3 Integrative perspective

The findings from the present bibliometric analysis reveal that macrophage-related peripheral nerve injury research has progressively evolved from the investigation of individual inflammatory mechanisms toward a multidimensional framework integrating cellular regulation, molecular mechanisms, and translational strategies. Early studies primarily focused on inflammatory activation and cytokine-mediated responses, whereas subsequent research emphasized macrophage polarization, Wallerian degeneration, and interactions between macrophages and Schwann cells, highlighting the importance of cellular coordination during nerve repair. More recent trends identified in this study further demonstrate a shift toward refined mechanistic analysis, including macrophage heterogeneity revealed by transcriptomics and single-cell RNA sequencing, subcellular regulation involving mitochondrial dysfunction and ion channel modulation, and regenerative strategies based on nerve conduits, exosomes, stem cells, and targeted delivery systems. Collectively, these findings suggest that macrophage-related peripheral nerve injury research is moving toward an integrated model in which macrophage states, local microenvironmental regulation, intercellular communication, and therapeutic intervention are considered as interconnected processes rather than independent research topics. The present study contributes to the field by systematically mapping the evolution of research themes, identifying emerging knowledge frontiers, and linking bibliometric patterns with potential biological mechanisms and future translational directions.

### 4.4 Limitations

This study has several limitations. First, regarding data sources, this study used only the Web of Science Core Collection and Scopus databases for literature retrieval. Although these databases provide broad and complementary coverage and standardized citation information suitable for bibliometric analysis, relevant studies indexed exclusively in other databases, such as PubMed, Embase, or regional databases, may have been missed. Because bibliometric indicators are dependent on database coverage and indexing practices, the observed publication output, collaboration networks, citation patterns, and research hotspots may therefore be influenced by database-related selection bias. Second, only English-language publications were included to maintain consistency in bibliographic metadata and facilitate standardized keyword cleaning and network analysis. However, this restriction may have excluded relevant studies published in other languages and may disproportionately represent research communities that publish pre-dominantly in English. Consequently, the findings of this study should be interpreted as primarily reflecting the macrophage-related peripheral nerve injury literature indexed in WoSCC and Scopus and published in English, rather than the entirety of global research activity in this field. The contributions of some countries, regional journals, research topics, or locally relevant research directions may therefore be underestimated. Finally, bibliometric analysis is mainly based on indicators such as publication output, citation frequency, keyword co-occurrence, and burst detection. Although these indicators can reflect research hotspots and developmental trends in the field, they cannot fully evaluate the experimental quality, level of evidence, or mechanistic reliability of individual studies. Future studies incorporating additional international and regional databases, multilingual literature, systematic reviews, and experimental validation may further improve the comprehensiveness, robustness, and generalizability of the conclusions.

## 5 Conclusion

In summary, this study used bibliometric tools, including VOSviewer, CiteSpace, and Bibliometrix, to systematically map the overall landscape of macrophage-related research in peripheral nerve injury over the past two decades. The results showed that research hotspots in this field have mainly focused on basic mechanisms, including macrophage polarization, Wallerian degeneration, neuropathic pain, macrophage–Schwann cell interactions, immunomodulation, and inflammatory microenvironment remodeling. In recent years, apoptosis and efferocytosis, mitochondrial dysfunction, ion channels, nerve conduits, mesenchymal stem cells, exosomes, and drug delivery have gradually emerged as new research hotspots. These findings not only deepen our understanding of the role of macrophages in the pathological progression and regenerative repair of peripheral nerve injury, but also provide new directions and potential targets for the development of therapeutic strategies for PNI. In addition, the application of single-cell RNA sequencing, spatial transcriptomics, and multi-omics integration is expected to further promote the analysis of macrophage heterogeneity, cell–cell interaction networks, and precise intervention targets, showing broad prospects for future application.

## Data Availability

The raw data supporting the conclusions of this article will be made available by the authors, without undue reservation.
