# Supplementary material for: Research hotspots and emerging trends of macrophages in peripheral nerve injury: a bibliometric and visualization analysis
Source: Front Cell Neurosci. 2026 Sep 17;20:1893849. doi: 10.3389/fncel.2026.1893849 (PMC13627019; doi:10.3389/fncel.2026.1893849)
Supplement: Supplementary file 1 [file Data_Sheet_1.docx]

Software_Analysis_Parameters

Country parameters

Organization parameters

Author parameters

Journal parameters

Keyword parameters

Journal dual-map overlay parameters

Citation burst parameters

R code
