# Supplementary material for: Research hotspots and emerging trends of macrophages in peripheral nerve injury: a bibliometric and visualization analysis
Source: Front Cell Neurosci. 2026 Sep 17;20:1893849. doi: 10.3389/fncel.2026.1893849 (PMC13627019; doi:10.3389/fncel.2026.1893849)
Supplement: Supplementary file 2 [file Table_1.docx]

Supplementary Table 1. Merging and modification records of country/region names and keywords.

| Standardization | Label | Replace by |
| --- | --- | --- |
| Country/Region | United States | USA |
|  | Peoples R China | China |
|  | England | United Kingdom |
|  | Scotland | United Kingdom |
| Keyword | axonal regeneration | axon regeneration |
|  | gene expression regulation | gene expression |
|  | sciatic nerve crush | sciatic nerve injury |
|  | immunoregulation | immunomodulation |
|  | Cells cultured | cell culture |
|  | sprague dawley rat | rat |
|  | dorsal-root ganglion | dorsal root ganglion |
|  | gene-expression | gene expression |
|  | peripheral-nerve injury | Peripheral nerve injury |
|  | schwann-cells | schwann cell |
|  | sciatic-nerve | sciatic nerve |
|  | spinal-cord | spinal cord |
|  | up-regulation | upregulation |
|  | c57bl mouse | mouse |
|  | mice | mouse |
|  | animals | animal |
|  | axons | axon |
|  | peripheral nerves | peripheral nerve |
|  | nerve injuries | nerve injury |
|  | macrophages | macrophage |
|  | schwann cells | schwann cell |
|  | cytokines | cytokine |
|  | chemokines | chemokine |
|  | disease models | disease model |
|  | humans | human |
|  | rats | rat |
|  | cells | cell |
|  | hydrogels | hydrogel |
|  | mechanisms | mechanism |
|  | procedures | procedure |
